# A pathway to sustainable development in China: The impact of local higher education expenditure on green total factor productivity

**DOI:** 10.1016/j.heliyon.2024.e34415

**Published:** 2024-07-09

**Authors:** Congying Ma, Yongxia Ma, Wei Wu

**Affiliations:** School of Humanities and Social Sciences, Beijing Institute of Technology, Beijing, 102401, China

**Keywords:** Local higher education expenditure, Green total factor productivity, Green technological innovation, Spillover effect, Influence mechanism

## Abstract

With the global warming crisis looming, the question of how to advance green total factor productivity (GTFP) has become an important concern confronting many developing countries. Although existing studies have demonstrated that total human capital can improve GTFP, the research has neglected to consider the influence of local higher education expenditure (LHEE), and no research has examined how LHEE spatially affect GTFP. Therefore, based on spatial economics theory, this study explores the spatial autocorrelation of LHEE and GTFP in China's 30 provinces from 2004 to 2021, employing a spatial Durbin model to analyze the spillover effect and influence mechanism of LHEE on GTFP. The results reveal that LHEE and GTFP exhibit positive global spatial autocorrelation. LHEE primarily improves GTFP and its subcomponents through spillover effects. The positive spillover effects in the three regions of China are significantly higher than the direct effects, whereas the direct effects in the eastern and central regions are positive but insignificant. Furthermore, LHEE promotes GTFP by advancing green technological innovation. The findings provide valuable insights to help policymakers address sustainable development goal 4 and develop synergistic regional GTFP growth policies to establish sustainable societies worldwide.

## 1 Introduction

Global warming, resource depletion, and environmental degradation are global challenges that require urgent attention. To manage increasing ecological risks, many countries have identified and implemented new ways to achieve sustainable development goals (SDGs) [1]. In this regard, green total factor productivity (GTFP) examines the efficiency of input and output indicators in a certain period within a country or region. GTFP is a comprehensive efficiency measure that simultaneously considers economic growth, energy consumption, and environmental pollution [2] and is considered a suitable strategic metric to concentrate on when seeking to achieve the SDGs [3]. Therefore, a consensus has been reached among major economies worldwide on ways to improve GTFP [4].

China's economy has soared since its reform and opening up, and it is now the world's second largest economy. However, in the wake of this rapid exponential development, China ranked 126^th^ out of 180 countries in the 2022 Global Environmental Performance Index, indicating challenges in the nation's ability to meet global SDGs [5]. GTFP growth is among the most prevalent topics in China [6]. Previous studies have explored the impact of industrial structure, energy consumption, technological innovation, and institutional design on GTFP [[6], [7], [8]]. SDG 4 indicates that higher education (HE) is important for advancing a nation's sustainable development [9], and some researchers have explored the effect of HE on GTFP, demonstrating that HE is correlated with improving GTFP by enhancing energy use efficiency and optimizing the energy structure [10,11]. Furthermore, HE is a source of innovative human capital that supports research and development (R&D) and technological advances [12], which subsequently impact environmental quality [4]. As a result, HE is likely a key factor in the future achievement of GTFP growth.

In the context of SDG 4, the Chinese government has also emphasized the crucial impact of HE, proposing a series of strategies for prioritizing the nation's educational development (e.g., popularizing HE and establishing world-class universities) [13]. In addition, China has the world's largest HE system and has achieved SDG 4 (Quality education) in 2022. In recent years, China has gradually invested in local HE (LHE), the main body of the HE system, for GTFP growth; for example, by cultivating dual-carbon talent, strengthening ecological civilization education, and building green campuses. However, several challenges remain such as insufficient LHE expenditure (LHEE), uneven regional LHEE, and an unclear understanding of the green influence of LHEE, which has been found to strongly affect GTFP efficiency and quality [13,14]. Therefore, in-depth research on the effect of LHEE on China's GTFP can provide a more thorough understanding of the contributions and influence mechanism of LHEE for advancing GTFP. The findings of this study can also help policymakers develop synergistic SDG 4–regional GTFP growth policies to promote sustainable societies worldwide.

Some research gaps remain in this field of inquiry. First, although previous studies have explored the effect of total human capital on GTFP, they have neglected to consider the influence of LHEE [[15], [16], [17]]. Notably, as the largest contributor to global carbon emissions, China's response is pivotal in worldwide green development [4]. Therefore, it is essential to examine the effect of LHEE on GTFP in China. Second, existing research has investigated the HE–GTFP nexus based on static analysis, ignoring the inherent spatial effect of LHEE on GTFP [18,19], which may produce inaccurate results. Spatial economics theory suggests that the spillover effect of education expenditure on GTFP growth is significant [20]. Because human capital is mobile between provinces, it can have an impact on local and surrounding areas, and the spillover effects of LHEE gradually strengthen with increased regional integration. Finally, previous research has been limited to examining the relationship between HE and GTFP, and influence mechanisms have not yet been systematically investigated [18,19].

To fill this gap, we propose the following research question. What spillover effects and impact mechanisms does LHEE exhibit concerning GTFP? Referencing spatial economics theory, we analyze the impact of LHEE on GTFP in China's 30 provinces from 2004 to 2021. We first investigate the spatial autocorrelation of LHEE and GTFP. We also employ a spatial Durbin model (SDM) to estimate the spillover effects of LHEE on GTFP and its subcomponents, green technological efficiency (GEC) and green technological progress (GTC). GEC represents the movement toward the frontier, while GTC represents the change in the production frontier [7]. We then conduct regional heterogeneity and temporal effects analyses, followed by an analysis of the transmission mechanism.

This study contributes to the literature in three ways. First, we incorporate spatial economics theory into the SDG framework, testing the potential power of LHEE through macro analyses of GTFP. The findings shift the paradigm of LHEE evaluation toward dimensions of social welfare in China. Second, we build a more accurate SDM to analyze the spillover effects of LHEE on GTFP and its subcomponents and conduct regional heterogeneity and temporal effects analyses. The novel results expand upon previous static research and are significant for guiding the flow of LHEE and promoting synergistic regional GTFP development that is tailored to local conditions. Third, we clearly reveal the influence mechanism of LHEE on GTFP to empirically inform policymakers when determining the best approaches to meet SDG 4 targets and achieve GTFP growth, deepening the existing research.

The remainder of the paper is structured as follows. Section 2 reviews the related literature and theoretical background. Section 3 describes the selection of variables and methods. Section 4 presents the results, and Section 5 presents further analyses. Section 6 tests the robustness of the results. Section 7 discusses the results, followed by the conclusions and policy implications. Section 9 includes a summary of the study's limitations and future research directions.

## 2 Literature review and theoretical background

### 2.1 Literature review

Advancing GTFP growth to cope with the global climate crisis has become a growing concern among scholars [10,11]. As SDG 4 proposes and research continues to investigate, endogenous growth theory affirms that human capital is crucial for a country to advance sustainable development [21]. Therefore, LHEE is a key approach for accumulating human capital and an important driving force for improving GTFP.

However, research on LHEE and GTFP has predominantly focused on total education-based human capital and GTFP. In China, education has been found to have an important influence on improving GTFP [[15], [16], [17]]. Similarly, Xiao and You [14] determined that human capital and investment in education have positive effects on GTFP efficiency. Furthermore, as a mediating variable, human capital also affects dependent variables (e.g., digital development and “information benefiting people” policies) influence on promoting GTFP [22,23]. However, one study reported that human capital has a significantly positive (but not significant) direct spatial spillover effect that restricts GTFP growth [24]. While human capital can have a strong positive direct effect, it does not exhibit any significant spillover effects, meaning that the increase in GTFP may be limited.

Notably, only a few studies have examined the impact of LHEE on GTFP. Yao et al. [18] found that increasing the number of graduate students and innovative human capital can improve GTFP in China, revealing a pattern of diminishing marginal benefits. Wang et al. [19] suggested that the effects of HE on GTFP are positive but insignificant. Employing spatial economics theory, one study demonstrated that HE promotes local GTFP but has no significant regional spillover effects, which was attributed to the unsmooth flow of human capital between provinces [25].

Moreover, some studies have explored the influence of HE on variables related to GTFP (e.g., green growth and carbon dioxide [CO_2_] emissions). Some researchers have asserted that HE has a significantly positive influence on green growth [26,27] and that the green effect of HE is greater than that of environmentally related technologies [28]. Furthermore, HE improves green growth through technological innovation, with a threshold effect in its impact [29]. Additionally, other studies have shown that HE can help reduce CO_2_ emissions [[30], [31], [32]]. Conversely, one study revealed that HE has no impact on improving green economic growth [33]. Some studies have also claimed that HE leads to increased pollution [34,35] and CO_2_ [36,37] emissions, and similar conclusions were reported [38,39] for the United States.

The rationale for these inconsistent findings is as follows. First, the results could be related to differences in sample selection and statistical methods and the presence of endogeneity or missing variables [40,41]. Specifically, statistical methods based on the spatial independence assumption neglect spatial spillover effects and can produce inaccurate results [20,42,43]. Second, a rapid increase in the scale of HE may have negative consequences (e.g., for energy consumption and pollutant emissions); however, advancing HE quality has a positive impact. Finally, investing in HE has long-term cumulative effects. HE disseminates environmental protection knowledge and changes individuals’ environmental practices, which can take a long time to materialize [10].

In summary, the literature provides a research foundation for this study, demonstrating that HE is a double-edged sword that can either promote green growth or damage the environment. However, several research gaps remain. First, most studies have explored the effect of total human capital on GTFP, ignoring the influence of LHEE. Furthermore, despite the development of spatial econometrics, the literature is limited in terms of its depth and has not yet estimated the spillover effect of LHEE on GTFP. More importantly, the few relevant studies have only verified the role of HE in GTFP and have not empirically examined the impact mechanism.

To fill these gaps, we analyze the impacts of LHEE on GTFP in China. We first construct a more accurate SDM model to estimate spatially lagged terms, including dependent and independent variables, which has statistical advantages compared with the spatial error model (SEM) and the spatial lag panel model (SLM) and alleviates the endogeneity problem caused by ignoring spatial lag [20]. Employing this model, we analyze the spillover effect of LHEE on GTFP and its subcomponents, followed by regional heterogeneity and temporal effect analyses. Finally, we estimate the transmission mechanism of LHEE on GTFP.

### 2.2 Theoretical background

LHEE can promote GTFP [44]. Endogenous growth theory has demonstrated that HE is crucial for advancing a country's sustainable development [21]. Specifically, investment in LHE can improve individuals' environmental knowledge and awareness and further affect their environmental practices [45]. LHE can also encourage the public to engage in greener lifestyle choices and honor environmental laws [46,47]. More directly, LHE is the main supplier of the green workforce and promotes sustainability policies, which advances local green development via green education and associated campus projects [11]. Universities worldwide are designing sustainability curricula and promoting practices for climate action to cultivate students that are capable of navigating the challenges of current and future changes in the world and meet the SDGs [48]. Therefore, we propose Hypothesis 1 as follows:

Hypothesis 1 LHEE promotes GTFP.

LHEE affects GTFP through the spatial spillover of education expenditure. Spatial economics theory indicates that education expenditure has positive externalities of knowledge spillover and talent mobility [21]. We further extend this assumption, asserting that the spatial effect of LHEE occurs through three channels. First, knowledge capital can move across neighboring provinces. For example, the pursuit of employment or business activities promotes the mobility of highly educated individuals between adjacent provinces, creating new knowledge and accelerating the interprovincial spillover of knowledge [49]. Second, highly educated individuals may immigrate across neighboring provinces (i.e., people may attend school in one province and live in a surrounding province). Third, LHE patterns may affect surrounding provinces due to demonstration, imitation, and competition effects (e.g., LHE cooperation and competition between provinces) [[50], [51], [52]]. Through spillover channels, the growth of GTFP in a specific province depends on the accumulation of LHEE in that province and the efficiency and quality of the spatial spillover of LHEE from neighboring provinces. Therefore, we propose Hypothesis 2 as follows:

Hypothesis 2 LHEE has a spillover effect on neighboring provinces' GTFP.

Green technological innovation (GTI) is a key mechanism by which LHEE influences GTFP [21]. Innovation ecosystem theory suggests that, as a key aspect of the national innovation system, LHEE is closely related to regional innovation development [29]. Most universities have established sustainability departments (e.g., dual-carbon professionals and laboratories of environmental sciences) to master environmental knowledge and promote the efficiency and quality of GTI via R&D and innovative human capital [53]. Endogenous growth theory suggests that GTI significantly reduces pollutant emissions and accelerates the promotion of green products, which can promote GTFP growth [21]. Therefore, improving GTI is considered to be a key strategy for addressing global warming and energy depletion [8,54]. We subsequently propose Hypothesis 3 as follows:

Hypothesis 3 LHEE promotes GTFP through GTI.

**Fig. 1:**
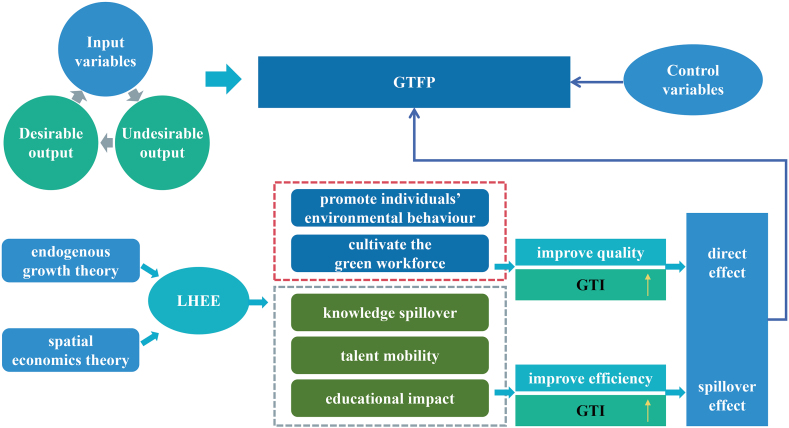
Theoretical framework.

Based on the above analysis, we illustrate the theoretical framework of this study in Fig. 1.

## 3 Data and methods

### 3.1 Variables

#### 3.1.1 Dependent variable

We use GTFP as the dependent variable and the GTFP index, which is measured employing a superefficient slack-based measure (super-SBM) model and the Malmquist‒Luenberger (ML) productivity index as a proxy variable. Considering environmental pollution and energy consumption, GTFP refers to the high-quality, innovative development of traditional TFP that integrates innovation-driven and green growth [6]. According to endogenous growth theory [21] and the production function under the neoclassical framework, capital, labor, and energy are the most basic innovation input elements. We use capital stock (K), labor input (L), and energy input (E) as input variables, and province-level real economic output (Y) as the desirable output and environmental pollution (C) as the undesirable output [[14], [15], [16]]. Table 1 presents the input–output variables used in this study.

**Table 1:** Input‒output variable selection and symbols.

Level-I indicators	Level-Ⅱ indicators	Level-Ⅲ indicators	Symbol
Input	Capital stock	Fixed assets investment	K
	Labor force	Employed persons	L
	Energy input	Electricity consumption	E
Desirable output	Economic output	Provincial real gross domestic product (GDP)	Y
Undesirable output	Environmental pollution	Industrial wastewater emissions	C
		Industrial sulfur dioxide (SO_2_) emissions	
		Industrial dust emissions	

#### 3.1.2 Independent variable

We select LHEE as the independent variable and local general HE per-student spending as a proxy variable. LHEE is a quasi-public product [55] that is primarily local government grants and is closely related to local governments' per capita fiscal revenue and the central government's financial subsidy policy. Moreover, LHEE is a key indicator for measuring sustainable development in LHE [56], which reflects the local government's emphasis on HE.

#### 3.1.3 Mechanism variable

We select GTI as a mechanism variable, using the number of green patent applications as a proxy [23]. According to endogenous growth theory [21], LHEE promotes GTFP through advancing technological innovation. Considering its inherently green and innovative characteristics, GTI can enhance energy efficiency, improve the energy consumption structure, and accelerate the transformation toward sustainable development [57]. GTI is also an important means of encouraging cleaner production and eco-innovation, which are important for addressing the contradiction between rapid economic growth and serious ecological concerns [58,59].

#### 3.1.4 Control variables

This study adopts government intervention (Gov), the level of openness to the outside world (Open), the informatization level (Inf), and the density of the railway network (Way) as control variables (Table 2).

(1) Gov: Government intervention, such as increasing financial support and attracting investment that can improve productivity and support GTFP growth are closely associated with GTFP [60]. However, excessive government intervention causes inefficiency in the market and has a negative impact on GTFP.
(2) Open: External openness can have a pollution haven or a pollution halo effect on the host country. Expanding the degree of openness can make it easier for a country to attract foreign direct investment and benefit from technological spillovers, which can improve local GTFP. However, developed countries may transfer energy- and pollution-intensive industries to such regions, negatively affecting the quality of the local environment and creating a pollution haven phenomenon in inflow areas [61].
(3) Inf: Information development promotes green production and improves resource allocation efficiency, which increases GTFP [22]. However, the flow of technological resources to a province can lead to a relative lack of resource support in other provinces, which hinders the overall sustainable development process.
(4) Way: Strengthening railway infrastructure construction enhances interprovincial connections and increases the proportion of green travel [56], enhancing GTFP. However, excessive railway construction may increase pollution emissions and negatively affect GTFP.

**Table 2:** Control variable selection and symbols.

Variable	Definition	Symbol	Unit
Government intervention	Local general public budget expenditure/provincial GDP	Gov	%
Level of openness to the outside world	Amounts of imports and exports of goods/provincial GDP	Open	%
Informatization level	Total postal and telecommunications business volume/provincial GDP	Inf	%
Density of the railway network	Mileage of railway network Operation/provincial area	Way	km/10^4^km^2^

### 3.2 Sample and data sources

This study uses panel data from China's 30 provinces (autonomous regions and municipalities directly under the control of the central government) from 2004 to 2021 as the research sample (excluding Tibet, Hong Kong, Macao, and Taiwan due to a lack of data). GTI data are obtained from the Chinese Research Data Services Platform. All other data are from the Educational Statistics Yearbook of China, the China Statistical Yearbook, the China Energy Statistical Yearbook, the China Environmental Yearbook, and provincial statistical yearbooks. According to the level and speed of economic growth, China is classified into east, middle, and west economic zones. Among these zones, the east is the richest and the west is the poorest [62]. All data are estimated at the 2004 price level, and GTFP, LHEE, Way, and GTI are in logarithmic form.

First, Table 3 presents the descriptive statistics of the data, revealing upward trends in GTFP and LHEE, which indicates that local governments are paying increasing attention to green and LHE development. Furthermore, GTFP and LHEE exhibit regional differences. The regional differences in GTFP are east > middle > west, indicating that GTFP is closely related to the level of local economic development. Furthermore, regional differences in LHEE are also east > middle > west, suggesting that LHEE is closely related to local governments' per capita fiscal revenue and the central government's financial subsidy policy.

**Table 3:** Descriptive statistics.

Variable	Obs.	Mean	SD	Min.	Max.
LnGTFP	540	0.787	0.162	0.449	1.749
LnLHEE	540	9.690	0.387	8.793	10.977
Gov	540	0.222	0.098	0.079	0.643
Open	540	0.301	0.361	0.008	1.721
Inf	540	0.065	0.045	0.014	0.290
LnWay	540	5.207	0.817	2.723	6.960
LnGTI	540	7.359	1.696	1.099	11.057

Table 4 shows that all variance inflation factors (VIFs) are lower than the critical value of 10, with no multicollinearity problems.

**Table 4:** Multicollinearity test.

Variable	VIF	1/VIF
LnLHEE	3.270	0.306
Gov	2.340	0.428
Open	2.070	0.484
Inf	1.290	0.774
LnWay	1.060	0.945
LnGTI	2.270	0.441

Table 5 demonstrates a correlation between variables, confirming that these variables are suitable for statistical analysis.

**Table 5:** Correlation analysis.

Variable	LnGTFP	LnLHEE	Gov	Open	Inf	LnWay
LnGTFP	1.000					
LnLHEE	0.452***	1.000				
Gov	0.013*	0.195***	1.000			
Open	0.070*	0.501***	−0.362***	1.000		
Inf	−0.006*	−0.008	0.410***	−0.107**	1.000	
LnWay	−0.031*	−0.009	0.111**	−0.072*	−0.106**	1.000

*p < 10 %, **p < 5 %, and ***p < 1 %.

Finally, theoretically, the dependent and independent variables are likely time persistent; therefore, it is necessary to conduct a stationarity test for the variables to avoid possible spurious regression. We use the panel unit root test, presenting the results in Table 6, confirming that the variables are stationary.

**Table 6:** Panel unit root tests.

Variable	ADF–Fisher statistic	LLC statistic
LnGTFP	5.984***	2.679***
LnLHEE	80.021**	−2.963***

*p < 10 %, **p < 5 %, and ***p < 1 %.

### 3.3 Method

#### 3.3.1 Super-SBM-ML model

According to the literature [63,64], the SBM model is the most effective method and widely used tool for assessing GTFP and has two advantages. (1) Imposing the weak disposability assumption reveals that decreasing undesirable outputs is costly, which is consistent with the actual process of production, and (2) compared with data envelopment analysis and the directional distance function approaches, which neglect input or output slack leading to biased estimation, the model introduces slack variables into the objective function to eliminate radial and oriented deviation and increase the accuracy and reliability of the estimated results [65]. It is shown in Equation (1):

(1) $$\begin{array}{l} \rho =\min\frac{1+\frac{1}{m}\sum_{i=1}^{m}\frac{s_{i}^{-}}{x_{n\times 1}^{t}}}{1-\frac{1}{q+l}\left[\sum_{r=1}^{q}\frac{s_{r}^{+}}{y_{rk}}+\sum_{p=1}^{l}\frac{s_{p}^{z-}}{z_{pk}}\right]}\\ s.t.\;\sum_{j=1,j\neq k}^{n}x_{ij}\lambda_{j}-s_{i}^{-}\geq x_{ik}\\ \sum_{j=1,j\neq k}^{n}y_{rj}\lambda_{j}+s_{i}^{+}\geq y_{rk}\\ \sum_{j=1,j\neq k}^{n}z_{pj}\lambda_{j}-s_{i}^{z-}\geq z_{pk}\\ \lambda ,s^{-},s^{+},s^{z-}\geq 0 \end{array}$$

where ρ denotes the target efficiency value; x_ij,_ y_rj,_ and z_pj_ are the input and output factors of the combination; m, q, and l denote the number of indicators for inputs, desirable outputs, and undesirable outputs, respectively; s (s^−^, s^+^, and s^z−^) indicate the slack in input and desirable and undesirable outputs, respectively; and z_n_ denotes the weight vector.

We adopt the ML productivity index model to examine the efficiency from period f to period f+1 [66], which is shown in Equation (2):

(2) $$ML_{t}^{t+1}=\sqrt{\frac{1+{\vec{D}}^{t}\left(x^{t},y^{t},z^{t},g^{t}\right)}{1+{\vec{D}}^{t}\left(x^{t+1},y^{t+1},z^{t+1},g^{t+1}\right)}\times \frac{1+{\vec{D}}^{t+1}\left(x^{t},y^{t},z^{t},g^{t}\right)}{1+{\vec{D}}^{t+1}\left(x^{t+1},y^{t+1},z^{t+1},g^{t+1}\right)}}=TEC\times TC$$

where ML represents GTFP growth rather than the degree of GTFP itself. We use the cumulative multiplier method to obtain GTFP, where if the GTFP of 2004 is 1, then, and so on. GTFP is decomposed into GEC and GTC, which are calculated in the same way. GEC change is primarily derived from the introduction of advanced management methods and concepts and changes in organization systems, while GTC change is predominantly derived from innovation and technology diffusion [7].

#### 3.3.2 Spatial econometric model

Both spatial economics theory and empirical results have shown that HE expenditure has spatial correlation characteristics [20,56]; therefore, it is essential to explore the spatial effects of LHEE on GTFP using a spatial econometric model.

First, we test the spatial correlation of LHEE and GTFP using the global Moran's Index (Moran's I) [20] as shown in Equation (3):

(3) $$I=\frac{n\sum_{i=1}^{n}\sum_{j=1}^{n}W_{ij}\left(X_{i}-\bar{X}\right)\left(X_{j}-\bar{X}\right)}{\sum_{i=1}^{n}\sum_{j=1}^{n}W_{ij}{\sum_{i=1}^{n}\left(X_{i}-\bar{X}\right)}^{2}}$$

where X denotes LHEE and GTFP, n is 30, and W_ij_ denotes the spatial geographic distance weight matrix. The Moran's I range is [−1,1]. A Moran's I value close to −1 or 1 indicates high global spatial autocorrelation.

W_ij_ is shown in Equation (4):

(4) $$W_{ij}=\left\{ \begin{array}{l} \frac{1}{{d_{ij}}^{2}},i\neq j\\ 0,i＝j \end{array}\right.$$

where W_ij_ denotes the geographic distance between the provincial capital cities of provinces i and j. If province i is closer to province j, then W_ij_ increases.

Then, based on the public investment economic growth model [67,68], we construct Equation (5) to calculate the impact of LHEE on GTFP:

(5) $${LnGTFP}_{it}=\beta_{1}LnLHEE_{it}+\beta_{2}Control_{it}+u_{i}+\nu_{t}+\varepsilon_{it}$$

Spatial econometric models include spatial autoregression (SAR), SEMs, and the SDM. SAR models measure the degree of spatial dependence between dependent variables, SEMs estimate the spatial interaction of error terms, and the SDM subdivides the sources of spatial lag into independent and dependent variables. Based on Equation (5), the three models are defined in Equations (6), (7), (8) as follows:

(6) $$SAR:{LnGTFP}_{it}=\rho \sum_{j=1}^{n}W_{ij}LnGTFP_{it}+\beta_{1}LnLHEE_{it}+\beta_{2}Control_{it}+u_{i}+\nu_{t}+\varepsilon_{it}$$

(7) $$SEM:{LnGTFP}_{it}=\beta_{1}LnLHEE_{it}+\beta_{2}Control_{it}+\mu_{i}+\nu_{t}+\varepsilon_{it},\varepsilon_{it}=\delta \sum_{j=1}^{n}W_{ij}\varepsilon_{it}+\sigma_{it}$$

(8) $$SDM:\mathrm{Ln}\;{GTFP}_{it}=\rho \sum_{j=1}^{n}W_{ij}LnGTFP_{it}+\beta_{1}LnLHEE_{it}+\beta_{2}Control_{it}+\theta_{1}\sum_{j=1}^{n}W_{ij}LnLHEE_{it}+\theta_{2}\sum_{j=1}^{n}W_{ij}Control_{it}+u_{i}+\nu_{t}+\varepsilon_{it}$$

where Control is the set of previously introduced control variables (Gov, Open, Inf, and LnWay); ρ and θ denote the spatial lagging coefficients of variables; β denotes the regression coefficients of the variables; μ_i_, ν_t_, and ε_it_ are individual and time effects and random error term, respectively; σ_it_ is the random error term of ε_it_; and δ is the spatial lagging coefficient of the error term.

However, after considering spillover effects, the three models above cannot directly estimate the effects of LHEE on GTFP. Finally, taking the SDM as an example, we employ the partial differential method to calculate the effects of LHEE on GTFP more precisely while accounting for the spatial autocorrelation between the variables [42] as shown in Equations (9), (10), (11):

(9) $$Direct\;effects={\left[{\left(I_{N}-\rho W\right)}^{-1}\left(\beta_{k}I_{N}+\theta_{k}W\right)\right]}^{\bar{d}}$$

(10) $$Spillover\;effects={\left[{\left(I_{N}-\rho W\right)}^{-1}\left(\beta_{k}I_{N}+\theta_{k}W\right)\right]}^{\bar{rsum}}$$

(11) $$Total\;effects={\left[{\left(I_{N}-\rho W\right)}^{-1}\left(\beta_{k}I_{N}+\theta_{k}W\right)\right]}^{\bar{d}}+{\left[{\left(I_{N}-\rho W\right)}^{-1}\left(\beta_{k}I_{N}+\theta_{k}W\right)\right]}^{\bar{rsum}}$$

where I_N_ is an identity matrix, k denotes the k-th LHEE, and d and rsum represent two operators that estimate the mean diagonal element and the mean row sum of the nondiagonal elements of the matrix, respectively.

#### 3.3.3 Interaction term model

To clearly explain the relationship between LHEE and GTI as a mechanism variable, we use the SDM as an example to construct a model. We also use the SDM to estimate the influence mechanism of LHEE on GTFP to construct an interaction term model. The models are shown in Equations (12), (13):

(12) $${LnGTI}_{it}=\rho \sum_{j=1}^{n}W_{ij}\;\mathrm{Ln}\;GTI_{it}+\beta_{1}LnLHEE_{it}+\beta_{2}Control_{it}+\theta_{1}\sum_{j=1}^{n}W_{ij}\;\mathrm{Ln}\;LHEE_{it}+\theta_{2}\sum_{j=1}^{n}W_{ij}Control_{it}+u_{i}+\nu_{t}+\varepsilon_{it}$$

(13) $${LnGTFP}_{it}=\rho \sum_{j=1}^{n}W_{ij}\;\mathrm{Ln}\;GTFP_{it}+\beta_{1}LnLHEE_{it}+\beta_{2}LnLHEE_{it}\times LnGTI_{it}+\beta_{3}Control_{it}+\theta_{1}\sum_{j=1}^{n}W_{ij}\;\mathrm{Ln}\;LGHEE_{it}+\theta_{2}\sum_{j=1}^{n}W_{ij}LnLHEE_{it}\times LnGTI_{it}+\theta_{3}\sum_{j=1}^{n}W_{ij}Control_{it}+u_{i}+\nu_{t}+\varepsilon_{it}$$

where GTI is the mechanism variable. LnLHEE_it_ × LnGTI_it_ denotes the interaction term between LHEE and GTI. In Equation (13), β_2_ is the coefficient of the interaction term that denotes the degree to which LHEE adjusts the direct effect of GTI on GTFP, and ϴ_2_ denotes the degree to which the spillover effect is regulated [25]. The other variables are set as in previous equations.

## 4 Empirical analysis

### 4.1 Spatial correlation analysis

Table 7 reveals that LHEE and GTFP have significant positive spatial autocorrelation characteristics. The findings confirm that the two variables are not completely random and are affected by geographic neighbors as provinces with low values are geographic neighbors, as are those with high variable values.

**Table 7:** Global Moran's I of LHEE and GTFP.

Year	Moran's I		Year	Moran's I	
	LHEE	GTFP		LHEE	GTFP
2004	−0.020	–	2013	0.115***	0.132*
2005	0.110***	0.037*	2014	0.111***	0.145*
2006	0.097***	0.031*	2015	0.207***	0.134*
2007	0.149***	0.039*	2016	0.041***	0.113*
2008	0.137***	0.036**	2017	0.150***	0.075*
2009	0.191***	0.139*	2018	0.089***	0.085*
2010	0.037***	0.179**	2019	0.132***	0.077*
2011	0.176***	0.173**	2020	0.140***	−0.019
2012	0.040***	0.152**	2021	0.181***	−0.038

*p <10 %, **p <5 %, and ***p <1 %.

Specifically, the global Moran's I of LHEE presents an overall increasing trend, indicating that the degree of spatial autocorrelation between provinces is rising. The values exhibit a volatile downward trend between 2009 and 2012 and between 2015 and 2021. A reasonable explanation for this is that the central government provides financial subsidies to education-poor provinces in the central and western regions (e.g., National Medium- and Long-term Education Reform and Development Plan and Western Higher Education Revitalization Plan) [56], which may increase the degree of spatial heterogeneity of funding in geographically close provinces, resulting in a decrease in the degree of LHEE spatial correlation.

Moreover, the global Moran's I of GTFP presents an inverted U-shaped trend, with a downward trend after 2010, revealing that the spatial correlation of GTFP weakened during this period. A possible explanation for this finding is that China has strengthened the construction of an ecological civilization and the development of a green economy in recent years [19]. Due to resource endowments and location conditions, an increasing number of interprovincial differences in GEC and GTC have emerged, which may increase the degree of spatial heterogeneity of GTFP in geographically close provinces, diminishing the degree of GTFP spatial correlation.

### 4.2 Spillover effect of LHEE on GTFP

#### 4.2.1 Model selection test

The results of the spatial autocorrelation test reveal that LHEE and GTFP exhibit spatial spillover effects; therefore, the spatial econometric model is appropriate for this empirical investigation. Referencing LeSage and Pace [42] and Elhorst [43], the Lagrange multiplier (LM) and robust LM tests indicate that spatial correlation exists and the SDM is a suitable technique. We then employ likelihood ratio (LR) and Wald to test the SDM, finding that it cannot be degraded to the SEM or SLM. Moreover, Hausman tests determine that the SDM with fixed effects is the most reasonable model. Additionally, compared with the dynamic SDM (DSDM), the SDM is more stable and suitable and has been used in previous research related to human capital and GTFP [14,25,43]. Finally, we select the SDM as the baseline model, presenting all test results in Table 8.

**Table 8:** Test results of model selection.

Test	Nationwide	Eastern	Middle	Western
LM (err)	19.116 ***	0.185	3.410*	13.529***
Robust LM (err)	14.995***	0.288	5.030**	5.000**
LM (lag)	5.824**	0.101	0.030	42.219***
Robust LM (lag)	1.703	0.204	1.650	33.689***
LR (err)	51.80***	53.19***	21.36***	17.30***
LR (lag)	30.74***	36.50***	17.58***	6.22
Hausman test	29.13***	−6.04	−34.19	−208.02
Wald test (err)	38.47**	49.24***	18.39***	14.19**
Wald test (lag)	30.65***	37.73***	18.68***	6.21

*p <10 %, **p <5 %, and ***p <1 %.

#### 4.2.2 Spillover effect analysis

We construct the SDM based on spatial economics theory to analyze the spillover effects, as shown in Table 9.

**Table 9:** Spillover effect analysis.

Variable	GTFP		GEC		GTC	
	Direct effect	Spillover effect	Direct effect	Spillover effect	Direct effect	Spillover effect
LnLHEE	0.080** (0.031)	0.391** (0.070)	0.050** (0.023)	−0.094*** (0.034)	0.011 (0.015)	0.709*** (0.062)
Gov	−0.010*** (0.002)	−0.001 (0.004)	−0.006*** (0.001)	−0.004* (0.002)	−0.001 (0.001)	−0.004 (0.004)
Open	0.0003 (0.0003)	−0.0003 (0.001)	0.0003 (0.0002)	0.0002 (0.001)	0.0003* (0.0001)	−0.002 (0.001)
Inf	−0.002 (0.002)	−0.011 (0.010)	−0.001 (0.001)	−0.002 (0.004)	0.0002 (0.001)	0.0002 (0.009)
LnWay	0.001 (0.006)	−0.021 (0.024)	0.005 (0.004)	0.025*** (0.009)	−0.006** (0.003)	−0.093*** (0.022)
ρ	0.563*** (0.050)		0.219*** (0.071)		0.772** (0.024)	
N	540		540		540	
R^2^	0.210		0.204		0.792	
Log-L	1306.767		1306.767		1306.767	

*p <10 %, **p <5 %, and ***p <1 %.

ρ shows a significantly positive correlation, which also reveals that GTFP, GEC, and GTC have spatial autocorrelation and spillover effects. The results demonstrate that spatial correlation is a key factor for improving GTFP and its subcomponents through integration strategy. These observations have been supported by previous studies [19,54].

In terms of the coefficients, the significant direct effect of LnLHEE on LnGTFP is 0.080, and the significant spillover effect is 0.391. These results indicate that LHEE increases local GTFP and generates positive spillover effects on neighboring provinces, supporting Hypotheses 1 and 2. This study reveals that investing in LHE enhances the level of environmental awareness, accelerates green technology progress, and promotes industrial upgrading, which further improves GTFP [19]. Furthermore, the spillover effect is approximately five times greater than the direct effect, which indicates that it is the main channel through which GTFP is improved. The coefficients of the effect of LnLHEE on LnGEC and LnGTC reveal that GTC is the primary channel for the transmission of positive spatial effects. The role of LHEE in the GTFP is predominantly related to the growth of the GTC.

Some control variables also exhibit significant effects on GTFP and its subcomponents, revealing that educational funding and these control variables jointly have a key influence on affecting GTFP growth. Among these effects, the positive spatial spillover effects are the strongest.

#### 4.2.3 Regional heterogeneity analysis

Because of the enormous degree of regional disparity in resource endowments across China, the effects of LHEE on GTFP are clearly regionally heterogeneous. We employ the SDM to test regional heterogeneity, as shown in Table 10.

**Table 10:** Regional heterogeneity analysis.

Effect	Eastern	Middle	Western
Direct effect	0.027 (0.042)	0.132 (0.135)	0.142*** (0.027)
Spillover effect	0.446*** (0.081)	0.308** (0.159)	0.271*** (0.061)
Total effect	0.472** (0.095)	0.440*** (0.132)	0.414*** (0.068)
Control variables	YES	YES	YES
ρ	0.425*** (0.073)	0.389*** (0.081)	0.623*** (0.062)
N	198	144	198
R^2^	0.221	0.383	0.315
Log-L	1306.767	1306.767	1306.767

*p <10 %, **p <5 %, and ***p <1 %.

For the three regions, the total and spatial effects are significantly positive and stronger than the direct effects. Overall, the contributions of LHEE to GTFP are in the order of eastern > central > western regions. If education spending increases by 1 %, then neighboring provinces may experience 0.446 %, 0.308 %, and 0.271 % increases in the eastern, central, and western regions, respectively. Considering the spillover effect, a 1 % increase in LHEE may cause 0.472 %, 0.440 %, and 0.414 % increases in the eastern, central, and western regions, respectively.

Notably, the direct effects are positive but insignificant for the eastern and central regions. The possible rationale for this is as follows. LHE investment can enhance residents’ environmental awareness and improve GTI, which promotes local GTFP. However, as the quality of HE improves, the quality of life of highly educated talent also improves. Although high-quality living conditions have strict environmental requirements, luxury purchases and replacement frequency of new products rises [69], which can have a crowding-out effect on “clean” technologies. In addition, rapid economic growth in the eastern and central regions has led to strong demand for highly educated talent. The rapid expansion of LHE through upgrading and infrastructure advances may increase local energy consumption and environmental pollution [31].

#### 4.2.4 Temporal effect analysis

LHE development often takes a certain number of years, meaning that there are differences between the short- and long-term green effects of education investment. The short-term effect is primarily manifested as direct monetary investment, which can promote GTFP by establishing sustainable sectors and improving green technology innovation capabilities, while the long-term manifestation refers to cumulative effects such as increasing citizens’ environmental literacy to enhance GTFP. We employ the SDM to test the temporal effects of LnLHEE and LnLHEE_t−1_ on GTFP, as shown in Table 11.

**Table 11:** Temporal effect analysis.

Variable	Direct effect	Spillover effect	Total effect
LnLHEE	0.032 (0.041)	0.110 (0.133)	0.142 (0.145)
LnLHEE_t-1_	0.046 (0.038)	0.283** (0.114)	0.329*** (0.120)
Control variables	YES	YES	YES
ρ	0.532*** (0.053)	0.532*** (0.053)	0.532*** (0.053)
N	510	510	510
R^2^	0.229	0.229	0.229
Log-L	514.329	514.329	514.329

*p <10 %, **p <5 %, and ***p <1 %.

The results reveal that the three effects of current education investment (LHEE) are slightly lower than those of previous education investment (LHEE_t−1_), showing that the long-term spillover and total effects of education investment are more obvious than the other effects. One reasonable explanation for this finding is that the impact of LHEE on GTFP is not immediate but cumulative and it takes a long time for individuals to complete HE, in addition to time to increase their environmental awareness, practice green lifestyles, or find green jobs [56]. Therefore, local governments should make short- and long-term plans when investing in LHE and focus on the long-term green effects of education funding to maximize the impact of education spending on GTFP.

## 5 Mechanism analysis

To explore the influence path of LHEE on GTFP, this study analyses the influence of LHEE on GTI and interaction between LHEE and GTI. LnLHEE × LnGTI are centrally processed to eliminate bias resulting from multicollinearity. Table 12 presents the model results.

**Table 12:** Mechanism analysis.

Variable	GTI			GTFP		
	Direct effect	Spillover effect	Total effect	Direct effect	Spillover effect	Total effect
LnLHEE	0.503*** (0.085)	2.434*** (0.359)	2.937*** (0.380)			
LnLHEE × LnGTI				0.034*** (0.010)	0.121*** (0.036)	0.155*** (0.039)
Control variables	YES	YES	YES	YES	YES	YES
ρ	0.777*** (0.026)	0.777*** (0.026)	0.777*** (0.026)	0.520*** (0.051)	0.520*** (0.051)	0.520*** (0.051)
N	540	540	540	540	540	540
R^2^	0.407	0.407	0.407	0.171	0.171	0.171
Log-L	1.107	1.107	1.107	7776.263	7776.263	7776.263

*p <10 %, **p <5 %, and ***p <1 %.

The direct and spillover effects of LnLHEE on LnGTI and LnLHE × LnGTI on LnGTFP are significantly positive, indicating that LHEE can synergistically promote GTFP by regulating GTI, supporting Hypothesis 3. Specifically, investing in LHE can increase the accumulation of innovative human capital, which further improves the growth of green technologies. Moreover, with increased educational input, the industrial structure can be transformed and upgraded, which can enhance the use of renewable energy and green innovation proclivities [12]. More environmentally friendly products and clean technologies will emerge in the future [70]. Furthermore, increased education expenditure can alleviate the technology rebound effect and reduce energy consumption and carbon emissions levels.

## 6 Robustness tests

This study uses three methods to examine the robustness of the SDM, the results of which are presented in Table 13.

(1) Replacing the spatial weight matrix. The scale competition and spillover effects of education funding are more obvious among regions with spatial contiguity [13,71]. Therefore, we employ the following spatial contiguity weight matrix to evaluate the robustness of the model. It is shown in Equation (14):
  (14) $$W_{ij}=\left\{ \begin{array}{l} 1,d_{ij}＜d\\ 0,d_{ij}\geq d \end{array}\right.$$
  where W_ij_ denotes the spatial contiguity weight matrix. If the distance from province i to province j is less than d, then W_ij_ is 1. When the distance from province i to province j is greater than or equal to d, W_ij_ is 0.
(2) Lagging the independent variable. As it takes time for LHEE to impact GTFP and because the DSDM is unstable, we lag the independent variable to evaluate the robustness of the SDM.
(3) Replacing the dependent variable. We use the GTFP index to replace the logarithmic form of GTFP to evaluate the robustness of the model.

**Table 13:** Robustness tests.

Effect	(1)	(2)	(3)
	Replacing the spatial weight matrix	Lagging the independent variable	Replacing the dependent variable
Direct effect	0.112*** (0.030)	0.069** (0.032)	0.193** (0.096)
Spillover effect	0.411*** (0.047)	0.371*** (0.062)	1.003*** (0.187)
Total effect	0.523*** (0.050)	0.440*** (0.061)	1.195** (0.188)
Control variables	YES	YES	YES
ρ	0.448*** (0.048)	0.542*** (0.052)	0.485*** (0.055)
N	540	510	540
R^2^	0.141	0.234	0.174
Log-L	541.838	513.690	59.982

*p <10 %, **p <5 %, and ***p <1 %.

Column (1) in Table 13 shows the estimation results of the spatial contiguity weight matrix, Column (2) shows the estimation results of LHEE_t−1_, and Column (3) shows the estimation results of the GTFP index. The findings demonstrate that the direction and significance of the coefficients in Table 9, Table 13 are consistent, confirming that the baseline SDM results are robust.

## 7 Discussion

This study reveals that LHEE and GTFP exhibit global spatial autocorrelation. The results align with previous studies [56,71,72], indicating that HE and green economic policies in geographically adjacent provinces are interdependent. In addition, the spillover effect of LHEE is a common feature of the public services offered by local governments and occurs via the spillover of knowledge and the mobility of highly educated individuals. Such spillover can also be classified into positive and negative effects.

The results also demonstrate that LHEE has a significant positive effect on GTFP and its subcomponents and that GTC is the primary channel through which education expenditure promotes GTFP. In contrast to most studies that used total education-based human capital as the variable [[15], [16], [17]], we form a link between LHEE and the green economy, emphasizing the roles and influence mechanisms of LHEE as an independent variable in GTFP growth. These results are also supported by previous observations [26,27], further extending human capital into the SDG framework. Previous research on the effect of HE on GTFP and green growth has revealed mixed findings, and the COVID-19 pandemic may have exacerbated inequalities in educational access and educational achievement due to uneven educational resource allocation, which is not conducive to meeting SDG 4 [73,74]. However, strong evidence has confirmed that HE is a crucial factor for GTFP growth beyond being merely an institution for training [26]. Moreover, a country with a high level of education is more likely to meet the SDGs than a country with a low level of education [11].

Our novel evidence also shows that the effects of LHEE on GTFP are primarily achieved through spatial spillover effects. These results differ from previous research [25] due to the use of different samples and data. Furthermore, the spillover effects of LHEE on GTFP are significantly positive in all three regions analyzed. The findings emphasize the importance of the spatial effect of LHEE on regional GTFP, which strongly confirms the spatial clustering and healthy competition effects of education expenditure. Consequently, the promotion of targets for GTFP growth requires interregional and cross-regional collaboration, which can be realized through the cross-regional flow of human capital and their spatial interactions, positive externalities, and spatial links promoting emissions reduction through green production modes and lifestyles. The findings broaden the application of spatial economics theory in education and regional GTFP growth and provide additional possibilities for future research for investigating the spatial spillover effect of LHEE on GTFP. The findings can also inform policymakers concerning how to make regional development strategies more effective in considering the spillover effects of LHEE.

The findings also indicate that the long-term green effect of LHEE has a more significant positive correlation than the short-term effect. This result contradicts the conclusions of Su and Gao [33], who found that HE increased CO_2_ emissions in the short and long term. This study further confirms the long-term effect of human capital accumulation. The role of HE in promoting GTFP growth is not only reflected in its short-term effects (e.g., industrial structure and GTI) but also in its long-term role in shaping individuals’ views and values or even altering their daily behaviors. Therefore, it is crucial to formulate long-term policies to achieve SDG 4.

The mechanism analysis demonstrates that education expenditure improves GTFP through GTI, consistent with the findings of a previous study [12]. Previous research has primarily concentrated on the effect of HE on GTFP or green growth, neglecting mechanism discussions [18,19]. This study introduces GTI into the research framework, which expands the research on HE, GTI, and GTFP. The results provide empirical evidence to illuminate their interaction, and the findings also reveal a specific path through which HE can promote GTFP growth.

## 8 Conclusions and policy implications

Based on spatial economics theory, we test whether LHEE and GTFP are spatially correlated and further investigate the spillover effects of LHEE on GTFP by estimating a SDM with fixed effects, followed by regional heterogeneity and temporal effect analyses and exploring the transmission mechanism.

The results reveal that (a) LHEE and GTFP exhibit significantly positive global spatial autocorrelation. Moreover, (b) LHEE primarily promotes GTFP and its subcomponents through the spillover effect. GTC is found to be the main way in which education spending can boost GTFP. (c) LHEE has significantly positive spillover effects on regional GTFP, which are greater than the direct effects in the three regions, while the direct effects are positive but insignificant in the eastern and central regions. (d) The long-term effect of LHEE on GTFP is more significantly positively correlated than the short-term effect. Furthermore, (e) LHEE enhances GTFP through GTI.

According to the above results, this study proposes three policy implications, which not only applies to China but also to other countries (e.g., those shifting from traditional development to GTFP development).

First, investing in LHE can promote China's high-quality GTFP growth. Therefore, local governments should increase financial support for LHE while exploring and promoting a human capital allocation structure in alignment with local GTFP growth. In addition, local governments should strengthen cooperation with local universities, incorporate green education in HE systems (e.g., disciplines, scholars, and university leaders), and go beyond considering it an interesting concept [75]. Specifically, local governments can encourage universities to create a “green curriculum,” pursue GTI, conduct research projects concerning sustainable development, and encourage students to participate in projects related to global development [44,47], which will advance the implementation of green education while meeting SDG 4.

Second, the sustainable development of LHEE aligns with the goal of GTFP growth; therefore, China's central government should establish a sustainable financial subsidy mechanism to ensure the fair distribution of education expenditures in different regions [76]. In addition, local governments should establish a sustainable regional investment mechanism for LHEE to promote sustainable GTFP growth. Regions with less investment in education and insufficient funding sources (e.g., the central region of China) should consider expanding the sources of funding and actively introducing social funds, which can ease the financial pressure placed on the government while promoting the development of education.

Finally, policymakers should consider the spillover effects of neighboring provinces when developing regional development policies, and local governments should strengthen cooperation and human capital exchange to improve the spillover and diffusion effects of funds. Furthermore, local governments can actively establish regional HE resource clusters and green innovation environments to promote regional GTFP connections, regional integration, and green development. For instance, the positive spillover effect of LHEE on regional GTFP can be fully utilized to improve the GTFP growth of regions where the direct effects are positive but not significant (e.g., eastern and central China). This situation ultimately narrows the degree of regional disparity and promotes the sustainable development of regional GTFP.

## 9 Limitations and future research directions

This study has some limitations that can be addressed in future studies. First, we consider the SDM a baseline model due to its applicability and stability. It would be interesting for future work to optimize the indicators to construct a stable DSDM and estimate the short- and long-term spillover effects of LHEE on GTFP. Second, future analyses can measure LHEE using different indicators to examine their impact on GTFP; for example, those related to LHE capital and enrolment in tertiary education. Third, we use GTI as a mechanism variable in this study, revealing an important channel through which to explain the relationship between LHE and GTFP. Future research can explore other mechanism channels such as information and communications technology, industrial structure, or energy consumption structure. Furthermore, future analyses can empirically explore how COVID-19 affected the influence of LHEE on GTFP since some studies have demonstrated that COVID-19 diminished the promotion of SDG 4 [73,74]. Finally, the study data are limited to China; therefore, future research could expand to and compare additional countries.

## Data availability statement

Data associated with the study has not been deposited into a publicly available repository. Data will be made available on request.

## CRediT authorship contribution statement

**Congying Ma:** Writing – review & editing, Writing – original draft, Validation, Supervision, Software, Methodology, Investigation, Formal analysis, Data curation, Conceptualization. **Yongxia Ma:** Writing – review & editing, Writing – original draft, Visualization, Validation, Investigation, Formal analysis, Conceptualization. **Wei Wu:** Writing – review & editing, Writing – original draft, Conceptualization.

## Declaration of competing interest

The authors declare that they have no known competing financial interests or personal relationships that could have appeared to influence the work reported in this paper.
